# Multivariate Statistical Approach to Image Quality Tasks

**Published:** 2018

**Authors:** Praful Gupta, Christos G. Bampis, Jack L. Glover, Nicholas G. Paulter, Alan C. Bovik

**Affiliations:** 1 Department of Electrical and Computer Engineering, The University of Texas at Austin;; 2 Netflix Inc.;; 3 National Institute of Standards and Technology;

**Keywords:** image quality assessment, generalized contrast normalization, multivariate statistical modeling, X-ray images

## Abstract

Many existing Natural Scene Statistics-based no reference image quality assessment (NR IQA) algorithms employ *univariate* parametric distributions to capture the statistical inconsistencies of bandpass distorted image coefficients. Here we propose a *multivariate* model of natural image coefficients expressed in the bandpass spatial domain that has the potential to capture higher-order correlations that may be induced by the presence of distortions. We analyze how the parameters of the multivariate model are affected by different distortion types, and we show their ability to capture distortion-sensitive image quality information. We also demonstrate the violation of Gaussianity assumptions that occur when locally estimating the energies of *distorted* image coefficients. Thus we propose a generalized Gaussian-based local contrast estimator as a way to implement non-linear local gain control, that facilitates the accurate modeling of both pristine and distorted images. We integrate the novel approach of generalized contrast normalization with multivariate modeling of bandpass image coefficients into a holistic NR IQA model, which we refer to as multivariate generalized contrast normalization (MVGCN). We demonstrate the improved performance of MVGCN on quality relevant tasks on multiple imaging modalities, including visible light image quality prediction and task success prediction on distorted X-ray images.

## 1. Introduction

The perceptual quality assessment of visual media has drawn considerable attention in the recent past owing to the millions of images and videos captured and shared daily on social media websites, such as Facebook, Twitter and Instagram. Large scale video streaming services such as YouTube, Netflix and Hulu contribute heavily to internet traffic, which continues to expand rapidly as consumer demand for content increases. Reliable assessment of picture quality by large groups of human subjects is an inconvenient, time-consuming task that is very difficult to organize at scale. Thus, objective no-reference (NR) image quality assessment (IQA) models, which do not require any additional information beyond the input image, are often deployed in such settings to automatically and accurately predict visual quality as perceived by an average human subject. These models have also been successfully used to perceptually optimize the image capture process to improve the perceptual quality of the acquired visual signals. In addition, ‘quality-aware’ perceptual strategies are used to compress visual media to deliver high quality content to consumers over constrained network bandwidths [1].

Many NR IQA algorithms have been proposed recently, which for increased clarity, we will broadly classify into three categories. 1) Distortion-specific approaches that include algorithms that predict the quality of images afflicted by one or more known distortion types such as blockiness [2], ringing [3] and blur [4,5] artifacts. These models are difficult to generalize to other distortion types. 2) Purely data-driven approaches involve the extraction of low-level image features such as color and texture statistics [6], which are then mapped to subjective image quality scores using regression. More recently, deep learners have been trained to learn large sets of low level image features, which are then used to feed classical regressors that map the features to subjective quality space [7]. The general framework of convolutional neural network-based IQA models involve feeding a pre-processed patch to convolutional layers, which are often followed by pooling layers. The learned features are then fed to a combination of fully connected layers followed by non-linear activation and dropout layers [8,9]. 3) Natural scene statistics (NSS)-based approaches leverage statistical models of natural images and quantify the severity of distortion by measuring the degree of ‘unnaturalness’ caused by the presence of distortions. The perceptual image quality is measured as a distance of the distorted image from the subspace of natural images [10–13].

A number of techniques have been devised for general purpose NR IQA. The generalized Renyi entropy and normalized pseudo-Wigner distribution have been used to model directionality or anisotropicity of the variance of expected entropy to predict image quality [14]. NSS-based models have been designed to extract quality-aware features under natural image models in the wavelet [13], spatial [12] and discrete cosine transform (DCT) domains [15], achieving high correlations with human opinion scores.

The divisive normalization transform (DNT), which is used to model the nonlinear response properties of sensory neurons, forms an integral component in the density estimation of natural images [16]. A commonly used parametric form of DNT is:

(1) $$y_{i}=\gamma \frac{x_{i}^{\alpha }}{\beta^{\alpha }+\sum_{j}x_{j}^{\alpha }}$$

where *x*_*i*_ denotes a natural image signal that has been processed with a bandpass linear filter, and {*α*, *β*, *γ*} are parameters that can be optimized on an ensemble of natural image data. As shown in [17], when bandpass natural images are subjected to DNT with *α* = 2 they become Gaussianized with reduced spatial dependencies. The underlying Gaussian Scale Mixture (GSM) [18] model of the marginal and joint statistics of natural (photographic) image wavelet coefficients also implies similar normalization (*α* = 2) of neighboring coefficients. In our recent work, we developed a generalized Gaussian Scale Mixture (GGSM) model of the wavelet coefficients of photographic images, including *distorted* ones [19]. This new model factors a local cluster of wavelet coefficients into a product of a generalized Gaussian vector and a positive mixing multiplier. The GGSM model demonstrates the hypothesis that the normalized wavelet-filtered coefficients of distorted images follow a generalized Gaussian behavior, devolving into a Gaussian if distortion is not present. A related approach was adopted in [20], where a finite Generalized Gaussian Mixture Model (GGMM) was used as a prior when modeling image patches in an image restoration task.

Here we build on the above ideas, and propose a generalized Gaussian-based local contrast estimator, which we use in conjunction with a multivariate density estimator to extract perceptual quality-rich features in spatial domain.

NSS models have been well studied on an increasing variety of natural imaging modalities, including visible light (VL), long wavelength infrared (LWIR) [21], fused VL and LWIR [22] and X-ray images [23]. This kind of statistical modeling of these imaging modalities has led to the development of new and interesting applications, and are of significance to the design of visual interpretation algorithms. In a like vein, here we explore the effectiveness and versatility of multivariate generalized contrast normalization (MVGCN) by deploying it in applications arising in two different imaging modalities. Specifically, blind quality assessment (QA) of VL images and the prediction of technician detection task performance on distorted X-ray images.

The rest of the paper is organized as follows. In Section 2, we describe the generalized contrast normalization technique, which forms the core of MVGCN. We detail the multivariate statistical image model in Section 3, and analyze the effects of distortions on the estimated parameters of the multivariate model. Section 5 describes the first application, whereby MVGCN features are used to predict the detection task performance of trained bomb experts on X-ray images. The second application is explained in Section 4, where the MVGCN model is used to drive an NR IQA algorithm. Finally, Section 6 concludes the paper with possible ideas for future work.

## 2. Generalized contrast normalization

It is well established in the vision science and image quality literature that processing a natural scene by a linear bandpass operation followed by non-linear local contrast normalization has a decorrelating and Gaussianizing effect on the pixel values of the images of these natural scenes [24–26]. This kind of processing of visual data mirrors efficient representations computed by neuronal processing that takes place along the early visual pathway. These statistical models of natural (photographic) images have been used effectively in applications ranging from low-level tasks such as image denoising [27–29] and image restoration [30,31] as well as higher level processes such as face recognition [32,33], object detection [34,35], and segmentation [36,37].

**Figure 1.**
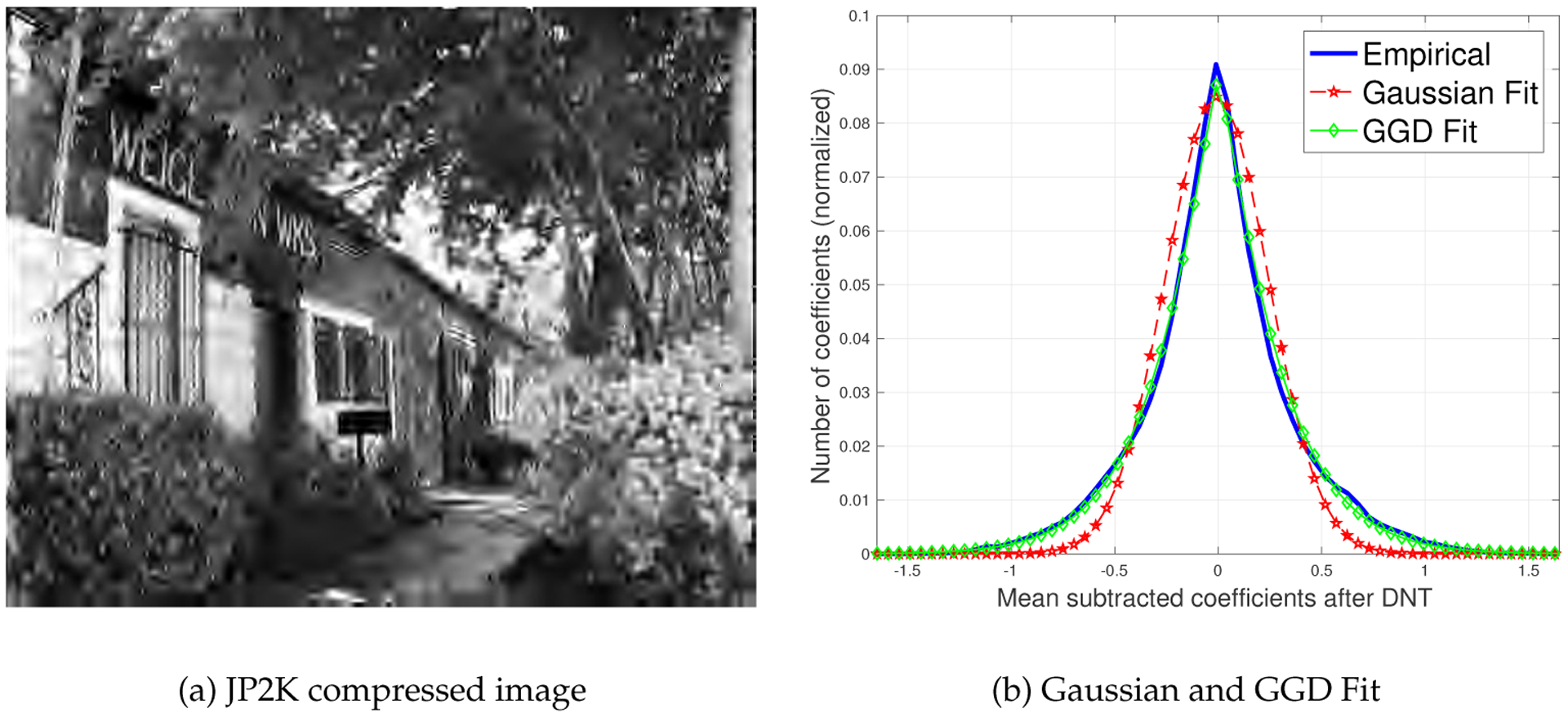
Histogram of divisively normalized bandpass coefficients of a JP2K compressed image. The difference between the best generalized Gaussian distribution (GGD) and Gaussian fits indicates that the generalized Gaussian-based contrast estimator is more appropriate for distorted coefficients. The computed Kullback-Leibler divergence values of the Gaussian and GGD fits were found to be KLD_gauss_ = 0.083 and KLD_GGD_ = 0.005 respectively.

A number of NSS-based IQA algorithms [12,13] operate under the hypothesis that the divisively normalized bandpass responses of a pristine image follow Gaussian behavior, and that the presence of distortion renders an image statistically unnatural, whereby the characteristic underlying Gaussianity is lost [17], as depicted in Fig. 1, where Gaussianity is a poor fit to the distribution of bandpass, divisively normalized coefficients of a JP2000 (JP2K) compressed image. Here we propose a way of collectively modeling both pristine and distorted images, using a generalized contrast normalization approach that is based on the premise that the divisively normalized bandpass coefficients of both distorted and undistorted images follow a *generalized* Gaussian distribution. We refer to the processed coefficients as mean subtracted generalized contrast normalized (MSGCN) coefficients.

Given a *M* × *N* grayscale image of intensity **I** = [*I*(*i*, *j*)], the MSGCN coefficients $\hat{I}=[\hat{I}(i,j)]$ are computed as:

(2) $$\hat{I}(i,j)=\frac{I(i,j)-\mu (i,j)}{\sigma_{gcn}(i,j)+C},$$

where ***μ*** = [*μ*(*i*, *j*)] and ***σ***_***gcn***_ = [*σ*_*gcn*_(*i*, *j*)] are the local weighted mean^1^ and local contrast fields defined as:

(3) $$\mu (i,j)=\sum_{k=-K}^{K}\sum_{l=-L}^{L}w_{k,l}I(i+k,j+l),$$

(4) $$\sigma_{gcn}(i,j)={\left((\gamma +\epsilon )\sum_{k=-K}^{K}\sum_{l=-L}^{L}w_{k,l}{\left|I(i+k,j+l)-\mu (i,j)\right|}^{\gamma }\right)}^{1/\gamma }$$

where *i* = 1, 2, …, *M*, *j* = 1, 2, …, *N* are spatial indices, and *w* = {*w*_*k*,*l*_|*k* = −*K*, …, *K*, *l* = −*L*, …, *L*} is a 2D isotropic Gaussian kernel normalized to unit volume with *K* = *L* = 3 and truncated to 3 standard deviations. *C* and *ϵ* are small positive constants used to prevent instabilities. *γ* is estimated using the popular moment-matching technique detailed in [38]. The generalized Gaussian corresponds to a Gaussian density function when *γ* = 2, and a Laplacian density function when *γ* = 1. MSGCN coefficients behave in a similar manner against different distortion types as do mean subtracted contrast normalized (MSCN) coefficients that are generated under the Gaussian model assumption (*γ* = 2)[12]. Distortions such as white noise tend to increase the variance of MSGCN coefficients, while distortions such as compression and blur, which increase correlations, tend to reduce variance. The MSGCN model is more generic than MSCN model and provides an elegant approach to study the statistics of distorted images.

## 3. Multivariate Image Statistics

**Figure 2.**
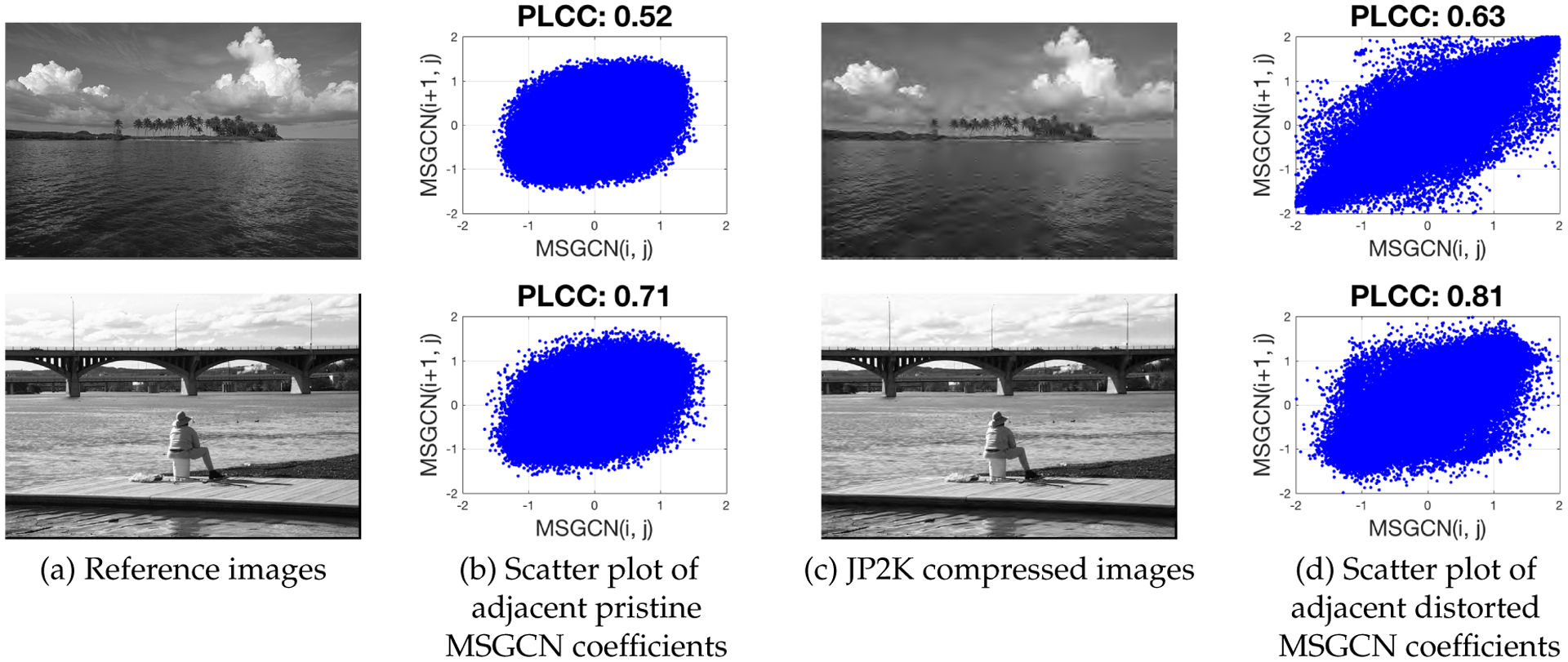
Illustration of the dependency of horizontally adjacent MSGCN coefficients of exemplar pristine images from the LIVE database [40]. The degree of these dependencies increases with the distortion severity. The PLCC^2^(Pearson’s Linear Correlation Coefficient) is used as a dependency measure.

In this section we use the aforementioned MSGCN coefficients to develop a multivariate NSS model and a way to extract quality-rich features. The generalized contrast normalization (GCN) transform is a form of local gain control mechanism that accounts for the non-linear properties of neurons, resulting from the pooled activity of neighboring sensory neurons [39]. These kinds of perceptually-relevant transformations account for the contrast masking effect, which plays an important role in distortion perception [39]. Although the GCN transform, as with other DNTs, reduces redundancies in visual data, the normalized coefficients of natural images may still exhibit dependencies in some form (depending on the image content), as depicted in Fig 2. Distortions such as compression, upscaling, and blur that reduce the amount of complexity of an image and that induce artificial correlations, tend to affect the MSGCN coefficients in a pronounced way. Increased statistical interdependencies are observed to occur between neighboring coefficients with increased distortion strength.

### 3.1. The Multivariate Generalized Gaussian Distribution

**Figure 3.**
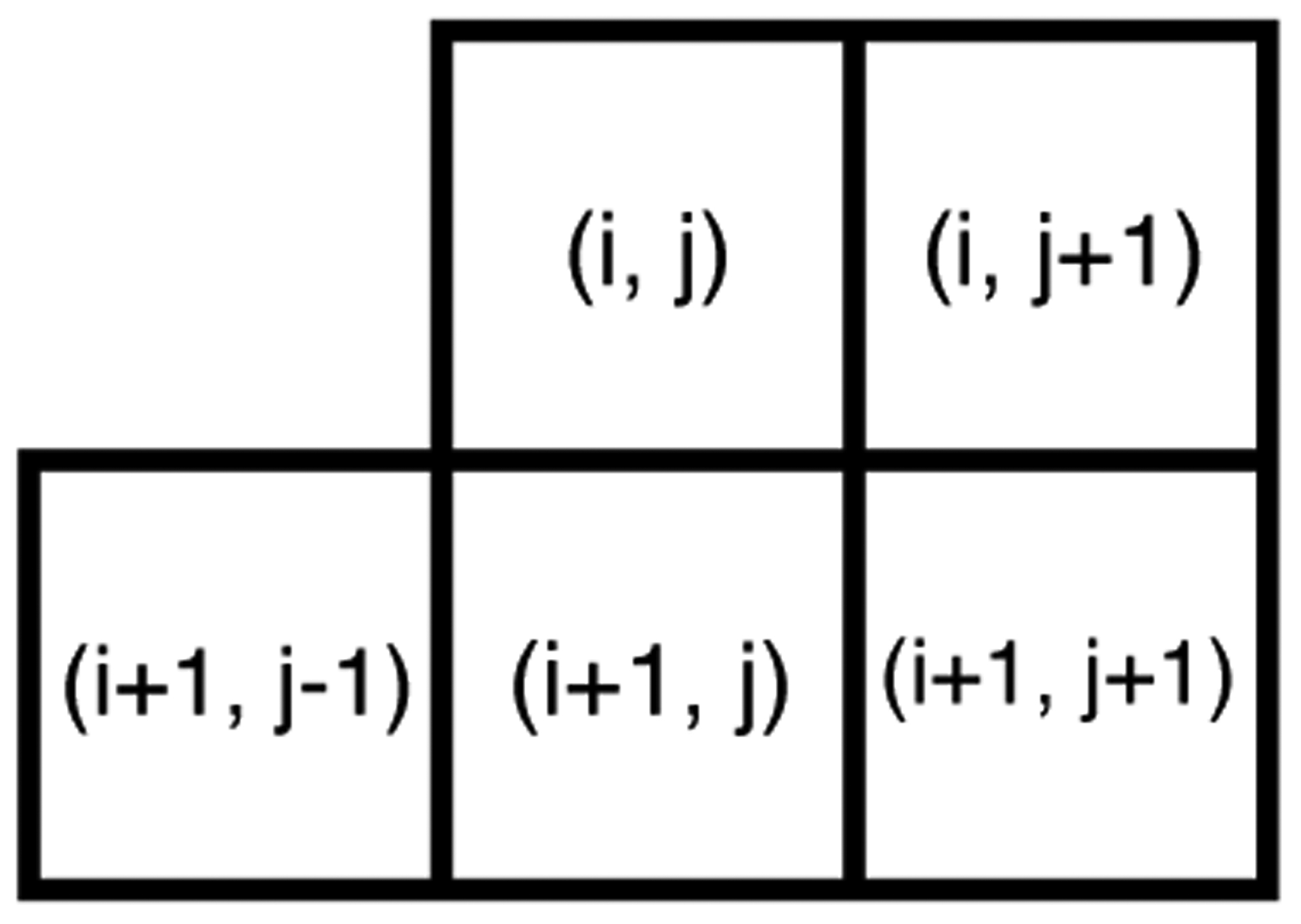
Set of adjacent MSGCN coefficients used to form the joint distribution model. The additional symmetrically placed samples relative to the coordinate (*i*, *j*) are not included to reduce the model size, and since it is likely that distortions along the same orientation will be redundant.

Once the MSGCN map of an input image is computed using (2), a 5D Multivariate Generalized Gaussian (MVGG) distribution is used to model the joint distribution of five neighboring coefficients as illustrated in Fig. 3. MVGG distributions have been extensively studied in the literature [41–44]. We utilize the Kotz-type distribution [41], which is a form of zero-mean multivariate elliptical distribution defined as:

(5) $$p_{x}(x)=\frac{\Gamma \left(\frac{d}{2}\right)\cdot s}{\pi^{d/2}\Gamma \left(\frac{d}{2s}\right)2^{d/2s}|\Sigma {|}^{1/2}}\cdot \text{exp}\left\{-\frac{1}{2}{\left(x^{T}\Sigma^{-1}x\right)}^{s}\right\},$$

where *s* is a shape parameter that determines the exponential fall-off of the distribution (the higher *s*, the lower the fall-off rate), Σ is the scale parameter (matrix) which controls the spread of the coefficients along different dimensions, *d* is the dimension of **x**, and Γ(·) is the gamma function

$$\Gamma (z)=\int_{0}^{\infty }e^{-t}t^{z-1}dt\;\forall z\geq 0.$$

The MVGG distribution becomes a multivariate Laplace distribution when *s* = 0.5, a multivariate Gaussian distribution when *s* = 1 and a multivariate Uniform distribution as *s* → ∞.

This form of MVGG distribution has also been used in a reduced-reference IQA framework [45], and in an RGB color texture model [46] of the joint statistics of color-image wavelet coefficients, a generalized Gaussian scale mixture (GGSM) model of the conditioned density of a GGSM vector [19], and in a no-reference IQA algorithm [15] to model the joint empirical distribution of extracted DCT features and subjective scores, where a bivariate version of the MVGG is used. The moment-matching scheme [41] used to estimate the shape and scale parameters of an MVGG is detailed in the Appendix.

### 3.2. Analysis of the Shape Parameter of the MVGG Distribution

**Figure 4.**
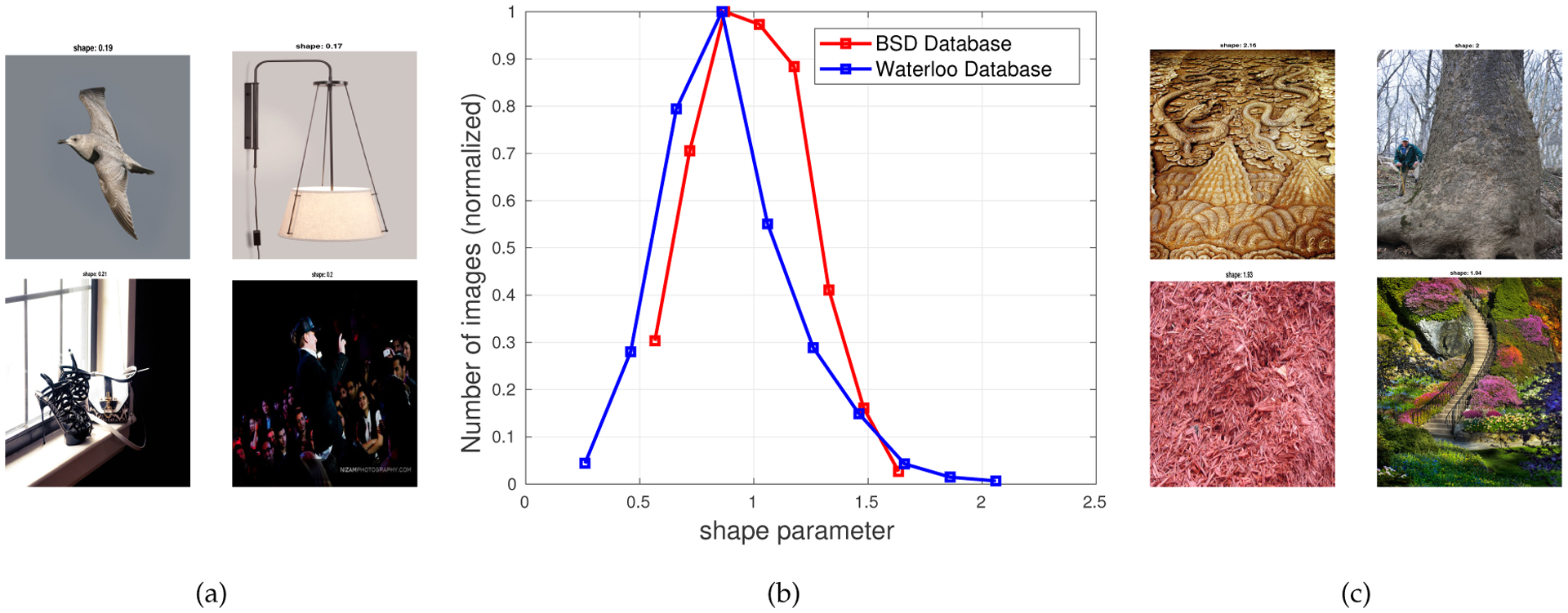
Empirical distribution (b) of the estimated shape parameter *s* obtained by fitting the joint MSGCN coefficients of pristine images from the Waterloo exploration and the BSD database with an MVGG. Images yielding values of *s* at extremities of the distribution (*s* ≤ 0.2 and *s* > 2.0) are shown in (a) and (c), respectively.

We next analyze how the shape parameter of the MVGG distribution varies when modeling the joint distribution of adjacent MSGCN coefficients of *natural*^3^, photographic images from two widely used databases – the Waterloo exploration database [47] and the Berkeley Segmentation Database (BSD)[48]. The Waterloo exploration IQA Database contains 4,744 pristine natural images reflecting a great diversity of real-world content. The Berkeley Segmentation Database was designed to support research on image segmentation and contains 300 training images and 200 test images. In our analysis, we only used ostensibly pristine images to generate MSGCN response maps, toward modeling a 5-dimensional joint empirical distribution of neighboring MSGCN coefficients using an MVGG density. Fig. 4(b) plots a histogram of the estimated shape parameter values of the MVGG model. The shape parameter peaked at around the same value (*s* = 1) on both databases, suggesting that the joint distribution of MSGCN coefficients of the pristine images may be reliably modeled as a multivariate Gaussian. This outcome may be viewed as a multivariate extension of the well-established Gaussian property of univariate normalized bandpass coefficients [18,24–26]. There are, however, a few samples within the studied collection of natural images where the estimated shape parameter deviated from *s* = 1. For example, a few images from the Waterloo exploration database, e.g., those shown in Fig. 4(a), contain predominantly flat, highly correlated regions which yielded peakier MVGG fits where *s* < 1. Cloudless sky regions (upper left of Fig. 4(a)) are bereft of any objects, and cause this effect. The lower two images of Fig. 4(a) have large saturated over/under-exposed areas, and may be viewed as substantially distorted. Overall, undistorted non-sky images of this type are rare. Conversely, the images shown in Fig. 4(c) are each almost entirely comprised of heavily textured regions, with less peaky fits (*s* > 1). These kinds of images are also unusual.

### 3.3. Effect of Distortions on the Shape Parameter

**Figure 5.**
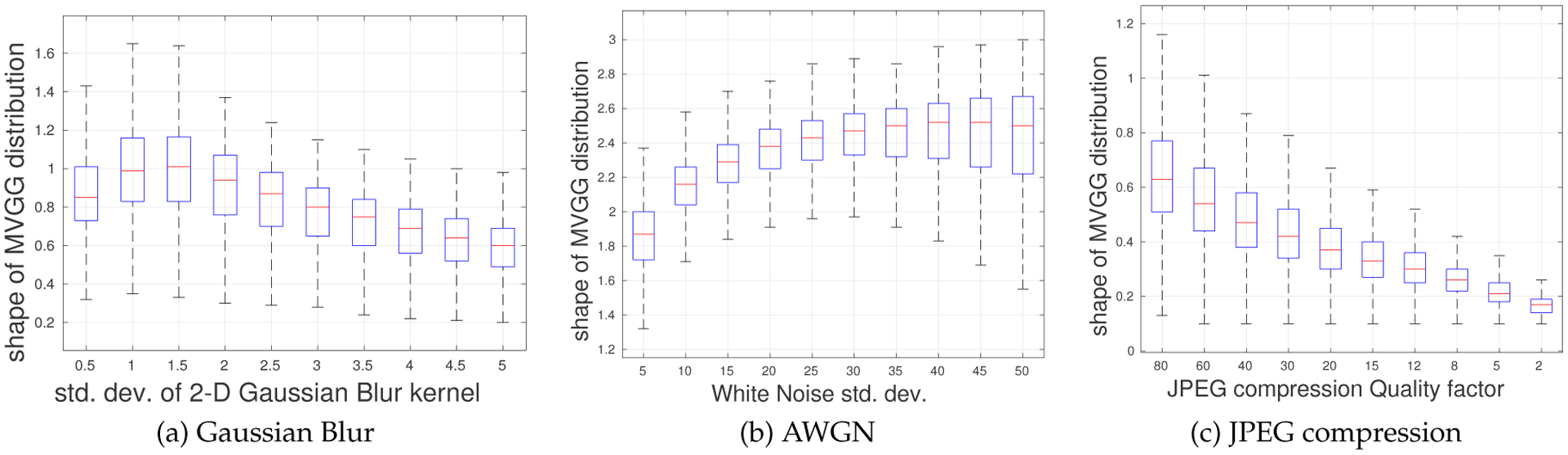
Boxplots of the estimated shape parameter *s* for three different distortions: (a) Gaussian blur, (b) AWGN and (c) JPEG compression^4^. Outliers were removed from the plots for better visualization.

Having established the relevance of the shape parameter of the MVGG and values it assumes on pristine images, we next examine how it behaves in presence of distortions. In this experiment, we degraded 1000 pristine images from the Waterloo exploration database using three common distortions – JPEG compression, Gaussian blur and additive white Gaussian noise (AWGN), each applied at ten different levels. We then followed a similar modeling procedure as that described in previous subsection – we fit the 5D empirical joint distribution of MSGCN coefficients of the distorted images with an MVGG distribution. Figure 5 depicts the way the shape parameter characteristically varies in the presence of the different degradation types and levels. Gaussian blur (Fig. 5(a)) and JPEG (Fig. 5(c)) degradations lead to peaky, heavy-tailed MVGG fits and reduced values of *s*. This effect becomes more pronounced with increasing distortion strength. Conversely, AWGN (Fig. 5(b)) degradations increase the randomness and entropy of an image, leading to larger values of *s*.

**Figure 6.**
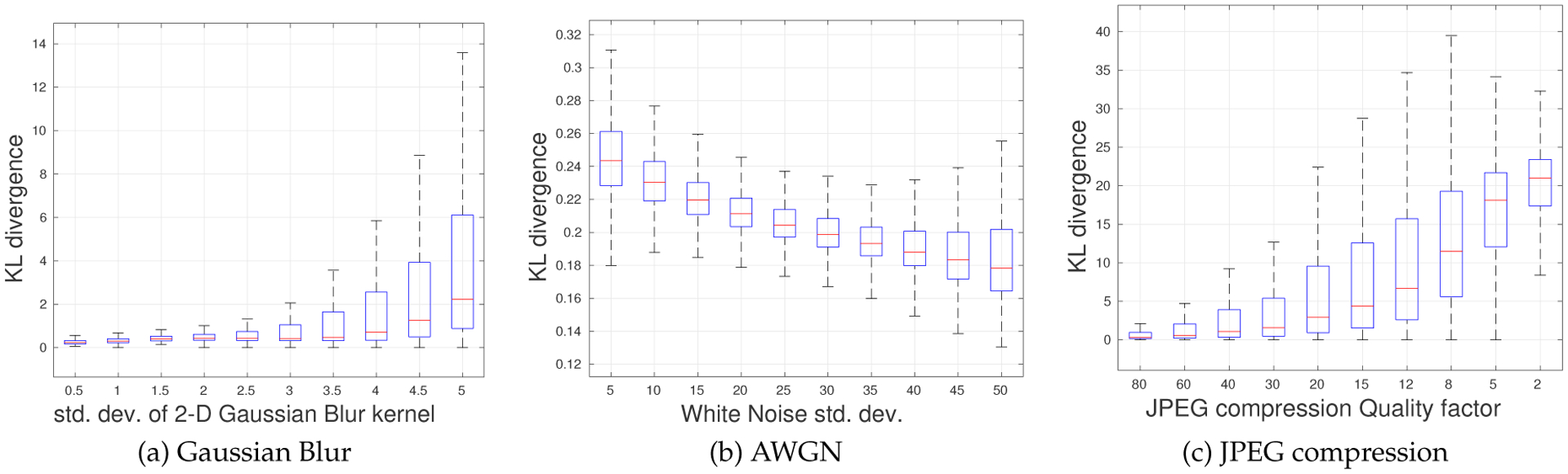
Boxplots of the KL divergence between the 2-D empirical distributions of MSGCN coefficients and their multivariate Gaussian fits for three different distortions: (a) Gaussian blur, (b) AWGN and (c) JPEG compression. Outliers were removed from the plots for better visualization.

The presence of some degradations deviate the distributions of distorted MSGCN coefficients from multivariate Gaussian behavior. To better understand this effect, we computed the Kullback - Leibler (KL) divergences between the empirical bivariate^5^ joint distribution of vertically adjacent MSGCN coefficients and its multivariate Gaussian fit, which are shown in Fig. 6. As shown in Fig. 6(b), increases of the AWGN standard deviation produced a slight decrease in the KL divergence, indicating that the joint distribution of the MSGCN coefficients becomes more similar to Gaussian, which is not unexpected given that the AWGN is Gaussian. Degradations such as blur and JPEG compression, which result in peakier MVGG fits, caused larger KL divergences, which increase with increasing distortion levels.

### 3.4. Feature extraction

Given that the MVGG model can be used to characterize distorted image statistical behavior well, we can build feature-driven image quality prediction tools. As a first set of ‘quality-aware’ features, compute the estimated shape parameter *s* and the five eigenvalues of the estimated covariance (scale) matrix Σ of the MVGG distribution. The premise behind the choice of these features is that the joint distribution of neighboring MSGCN coefficients of pristine images follow a multivariate Gaussian distribution, but the presence of distortion causes deviation from Gaussianity. Since each distortion affects the coefficient distributions in a characteristic manner, it is possible to predict the type and perceptual severity of distortions, and hence, the perceived image quality.

**Figure 7.**
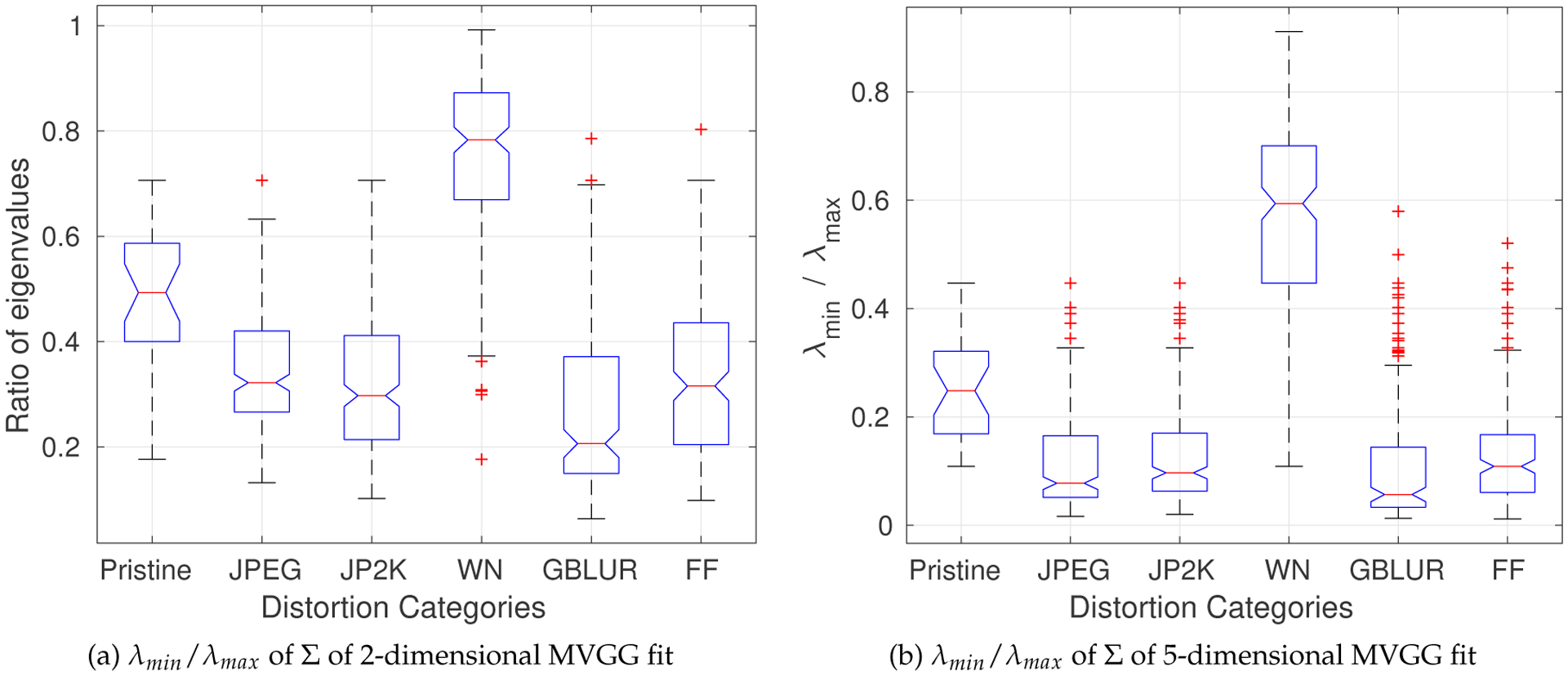
Boxplots of the ratio of the minimum and maximum eigenvalues of the estimated Covariance matrix Σ over all reference and distorted images from the LIVE database; “WN” is white noise, “GBLUR” is Gaussian blur and “FF” is fast fading Rayleigh channel.

As shown in Fig. 2, even after the application of the GCN transform, the MSGCN responses remain correlated on images degraded by correlation-inducing distortions such as compression and blur. Such distortions lead to more polarized eigenvalues of the estimated covariance matrix than do other distortions (AWGN). In order to demonstrate the effect of distortions on the eigenvalues, we use the ratio of the minimum and maximum eigenvalues (*λ*_*min*_/*λ*_*max*_) of the estimated scale matrix Σ from the best 2D MVGG fit to the vertically adjacent MSGCN coefficients. We also fit a 5D MVGG to the five neighboring coefficients (as shown in Fig. 3(b)). Figure 7 shows the boxplots of the ratio *λ*_*min*_/*λ*_*max*_ over all images from the LIVE database [40], but classified by distortion type. The pattern of variation of the eigenvalues of the estimated covariance matrix in the presence of different distortion types is indicative of the rich perceptual quality information captured by eigenvalues.

The pairwise products of adjacent MSGCN coefficients, like those of MSCN coefficients, also exhibit statistical regularities on natural, photographic images. We follow a similar modeling approach as that described in [12], and use a zero-mode asymmetric generalized gaussian distribution (AGGD) to fit the pairwise products along four directions whose density is defined as [12]:

(6) $$f\left(x;\alpha ,\sigma_{l}^{2},\sigma_{r}^{2}\right)=\{\begin{array}{l} \frac{\alpha }{\left(\beta_{l}+\beta_{r}\right)\Gamma \left(\frac{1}{\alpha }\right)}\text{exp}\left(-{\left(\frac{-x}{\beta_{l}}\right)}^{\alpha }\right),x<0\\ \frac{\alpha }{\left(\beta_{l}+\beta_{r}\right)\Gamma \left(\frac{1}{\alpha }\right)}\text{exp}\left(-{\left(\frac{x}{\beta_{r}}\right)}^{\alpha }\right),x\geq 0, \end{array}$$

where

$$\beta_{l}=\sigma_{l}\sqrt{\frac{\Gamma (1/\alpha )}{\Gamma (3/\alpha )}}$$

and

$$\beta_{r}=\sigma_{r}\sqrt{\frac{\Gamma (1/\alpha )}{\Gamma (3/\alpha )}}.$$

The AGGD parameters (*α*, *β*_*l*_, *β*_*r*_) are estimated using the moment-matching technique described in [49]. In addition to (*α*, *β*_*l*_, *β*_*r*_), AGGD mean $\mu =\left(\beta_{l}-\beta_{r}\right)\frac{\Gamma (2/\alpha )}{\Gamma (1/\alpha )}$ yields a fourth quality-aware feature. Extracting these four parameters along four orientations (*H*, *V*, *D*1 and *D*2) given by:

$$H(i,j)=\hat{I}(i,j)\hat{I}(i,j+1)\;V(i,j)=\hat{I}(i,j)\hat{I}(i+1,j)\;D1(i,j)=\hat{I}(i,j)\hat{I}(i+1,j+1)\;D2(i,j)=\hat{I}(i,j)\hat{I}(i+1,j-1),$$

where *i* ∈ {1, 2, 3, ..*M* − 1} and *j* ∈ {1, 2, 3, ..*N* − 1} are spatial indices, yields a total of 16 features.

In order to capture even higher-order correlations caused by complex distortions, we model the joint paired-product response map along the four directions (*H*, *V*, *D*1 and *D*2) using a 4-dimensional MVGG distribution. The eigenvalues of the estimated covariance matrix of the 4D MVGG density are extracted as an additional set of four quality relevant features. Since all of the features are extracted at two scales, a total of 26 × 2 = 52 perceptually-relevant quality-aware MVGCN features are computed. A brief summary of all of these features and their methods of computation is laid out in Table 1. In subsequent sections, we study the effectiveness of the MVGCN features by applying them to multiple image quality relevant tasks.

## 4. Quality Assessment of Visible Light images

In order to demonstrate the quality-rich feature extraction capabilities of the MVGCN model, we utilized them for the blind image quality assessment task. We compared the performance of MVGCN against a number of well-known NR IQA algorithms, such as SSEQ [50], CORNIA [51], CNN-IQA [8], BLIINDS [15], NIQE [10], BRISQUE [12] and DIIVINE^6^ [13] (all of which are publicly available), and two full reference (FR) IQA algorithms – PSNR and MS-SSIM [52]. We conducted our experiments on four widely used IQA databases namely: LIVE [40], TID08 [53], CSIQ [54] and LIVE in the Wild Challenge [55]. In all of the experiments, each model was trained on 80% of the database while the other 20% was used for testing. A support vector regressor (SVR) was used with radial basis function (RBF) to map quality features to the DMOS (Differential Mean Opinion Scores) after determining its parameters using 5-fold cross validation on the training set. The train-test splits were carried out in a manner to ensure that the training and test sets would not share reference images, so that the performances of the models would reflect their ability to learn distortions, without bias from overfitting on image content. A total of 100 such splits were performed, and the median Spearman’s rank ordered correlation coefficient (SROCC) and Pearson’s Linear correlation coefficient (PLCC) computed between the predicted quality scores and the DMOS are reported in Table 2. The overall results reported in Table 2 were computed by first applying Fisher’s z-transformation [56] given by:

(7) $$z=\frac{1}{2}\text{ln}\frac{1+r}{1-r},\text{ where }r\text{ is}\;\text{SROCC}\;\text{or PLCC},$$

and then averaging the transformed correlation scores for each method across each database, and finally applying the inverse Fisher’s z-transform.

Learning-based algorithms that involve a training stage to learn optimal parameters are sometimes susceptible to overfitting, especially when trained and tested on the same database, due to similar modeling of distortions, similar experimental conditions, and other factors. The main objective of NR IQA algorithms is their ability to generalize well on other datasets. To demonstrate the generalization capabilities, we trained the NR IQA models on one entire database and evaluated their performance on common distortion types from other databases, including: JPEG2000 (JP2K) and JPEG compression, Gaussian blur and AWGN. Table 3 reports the database-independence performance of MVGCN, while Table 4 compares its aggregate performance against other NR IQA models across four leading IQA databases. We used the non-parametric Wilcoxon rank-sum test to conduct the statistical significance analysis (reported in Table 5) between different algorithms across multiple databases. As can be noted from the tables, MVGCN performed better than several leading NSS-based NR IQA algorithms, and competed well against CORNIA [51], which uses raw image patches in an unsupervised manner to learn a dictionary of local descriptors. CORNIA extracts a 20000-D feature vector and is much more computationally expensive than MVGCN, as shown in the time complexity analysis results reported in Table 6. Although CNN-IQA performed better than other models on CSIQ and TID08 databases, it failed to deliver comparable performance on LIVE Challenge database, which consists of authentic real-world distortions. This raises questions on the practical application of such models and limits their use in real-world scenarios.

## 5. Predicting detection performance on X-ray images

In previous work, we studied the natural scene statistics (NSS) of X-ray images and found that the NSS modeling paradigm applies quite well to X-ray image data, although the model is somewhat different from that of visible light (VL) images [23,57]. In prior work, we used a nominal set of X-ray NSS features along with standardized objective image quality indicators (IQIs) to analyze the relationship between X-ray image quality and the task performance of professional bomb technicians who were asked to detect and identify a collection of diverse potential threat objects.

To analyze the effects of image quality on task performance, we conducted a human task performance study in which professional bomb technicians were asked to detect and identify improvised explosive device (IED) components^7^ in X-ray images that we created, degraded, and presented to them in an interactive viewing environment [58]. The degradations included spatially correlated noise, reduced spatial resolution, and combinations of these. The NIST-LIVE database of ground truth judgments of bomb experts was then used to evaluate the predictive performance of the objective X-ray image quality features. More details regarding the task performance study protocols can be found in [59].

Given that the MVGCN model provides a powerful NSS-based perceptual image quality feature extractor, we examined its performance against other NSS-based models and also against conventional IEEE/ANSI N42.55 [60] metrics. We hypothesized that the presence of degradations would change the characteristic statistical properties of the MSGCN coefficients of X-ray images, which would allow MVGCN model to better capture degradations, and would better correlate with the outcomes of expert detection and identification tasks conducted on degraded X-ray images.

The models used for comparison are the QUality Inspectors of X-ray images (QUIX) model [57], the IEEE/ANSI N42.55 standard [60] and combinations of these. QUIX features are a set of simple and efficient NSS-based perceptual quality features that accurately predict human task performance. In [57], QUIX considers only horizontal and vertical correlations while extracting features denoted as ‘*pp*’ features. In order to be consistent and to have a fair comparison against QUIX, we developed a reduced feature version of MVGCN, which we refer to as MVGCN-X-ray, which does not include the products of diagonal coefficients as part of the paired-product modeling and corresponding MVGG fits. A summary of the MVGCN-X-ray features used and the feature extraction procedure is described in Table 8.

Image quality indicators (IQIs) are a set of standard objective image quality metrics defined in IEEE/ANSI N42.55 [60]. These IQIs are determined by analysis of images of a standard test object under test conditions. In our analysis, we used eight IQIs, including ‘steel penetration’, ‘spatial resolution’, ‘organic material detection’, ‘dynamic range’, ‘noise’, and three other descriptive features that are extracted from the spectral distribution of the measured modulation transfer function (MTF), noise equivalent quanta (NEQ) and noise power spectrum (NPS).

Given that CORNIA is among the top performing IQA algorithms, albeit much more computationally expensive, as observed in the previous application, we compared its time complexity against MVGCN on X-ray images. CORNIA required about 50 times more time than MVGCN-X-ray did (as reported in Table 7) to extract features from high spatial-resolution^8^ X-ray images.

To evaluate performance, we divided the NIST-LIVE database on the basis of component and clutter combinations. The component categories include IED components: ‘power source’, ‘detonator’, ‘load’, ‘switch’ and ‘metal pipe’, which are labeled by professional bomb technicians, if *found* in an image, else labeled as *not found*. Here we consider the task of measuring the accuracy of objective image quality models to predict the detection performance of experts. We further divided each category into four clutter types: clutter (laptop), shielding (steel plate), clutter with shielding, and no clutter. Clutter/Shileding was added to some images to make the detection task more challenging.

We then devised a binary classification framework whereby features were mapped to a binary variable indicating whether the component was successfully identified by an expert. We used a logistic regression model to be consistent with [57]. The data from each component-clutter category was divided into an 80 % training set to learn logistic function parameters, which were then used to predict on the remaining 20 % test set. We used a similar performance evaluation methodology as followed in [57] – generated random disjoint train-test splits and computed median log loss and area-under-the-ROC-curve (AUC) scores over 1000 iterations (reported in Table 9).A smaller value of log loss and a larger value of AUC indicates superior classification performance, implying better correlation with human judgments.

We also demonstrated in [57] that QUIX features and IQIs supply complementary information, which when combined into a single predictor performed better than either of them in isolation. Under a similar premise, we augmented MVGCN-X-ray features with IQIs to obtain similar benefits in performance. As shown in Table 9, the combination of MVGCN-X-ray with IQIs yielded better performance than any of the other features in isolation, while competing well against the combination of QUIX and IQIs. The improvement in performance of the combination can be attributed to the capture of different levels of distortion-sensitive higher-order correlations by the MVGCN-X-ray features and by complementary X-ray image quality information supplied by IQIs.

## 6. Conclusion

We designed a multivariate approach to NR IQA which uses generalized contrast normalization – a form of DNT that is more suitable to model degraded image coefficients. We investigated the effect of degradations on the estimated shape and eigenvalues of the estimated covariance matrix of MVGG fit to the joint distribution of neighboring MSGCN coefficients. Further, we demonstrated applications of the MVGCN model to the blind QA of visible light images and on the prediction of threat object detection and identification by trained experts on degraded X-ray images, achieving near state-of-the-art performance in both applications.

There are a number of possible future directions. It is of interest to utilize the MVGCN model to design a spatio-temporal model of normalized bandpass video coefficients for video QA. The aforementioned multivariate modeling approach is also possibly extensible to other NSS models that utilize univariate parametric distributions of bandpass image coefficients. Furthermore, studying the statistics of other imaging modalities such as millimeter-wave, computed tomography (CT), and multi-view X-ray images are also potential future directions of exploration.

## Figures and Tables

**Table 1.** Feature summary of joint MSGCN(*m*), paired-products (*pp*) and joint paired-products (*j*) coefficients. All features are extracted at two scales..

Feature ID	Feature Description	Computation Procedure
*m*_1_	shape	5-D MVGG fit to MSGCN coefficients
*m*_1_ - *m*_5_	eigenvalues of scale matrix	5-D MVGG fit to MSGCN coefficients
*pp*_1_ - *pp*_4_	shape, mean, left variance and right variance	AGGD fit to H pairwise coefficients
*pp*_5_ - *pp*_8_	shape, mean, left variance and right variance	AGGD fit to V pairwise coefficients
*pp*_9_ - *pp*_12_	shape, mean, left variance and right variance	AGGD fit to D1 pairwise coefficients
*pp*_13_ - *pp*_16_	shape, mean, left variance and right variance	AGGD fit to D2 pairwise coefficients
*j*_1_ - *j*_4_	eigenvalues of scale matrix	4-D MVGG fit to H, V, D1 and D2 pp coefficients

**Table 2.** Median Spearman’s Rank Ordered Correlation Coefficient (SROCC) and Pearson’s Linear Correlation Coefficient (PLCC) across 100 train-test trials on the LIVE, CSIQ, TID08 and LIVE Challenge databases. The best two NR IQA models are boldfaced.

DB	LIVE		TID08		CSIQ		Challenge		Overall	
	SROCC	PLCC	SROCC	PLCC	SROCC	PLCC	SROCC	PLCC	SROCC	PLCC
*PSNR*	*0.892*	*0.883*	*0.561*	*0.571*	*0.803*	*0.800*	-	-	*0.756*	*0.758*
*MS-SSIM*	*0.953*	*0.942*	*0.860*	*0.845*	*0.913*	*0.896*	-	-	*0.894*	*0.888*
SSEQ	0.889	0.889	0.635	0.680	0.691	0.749	0.476	0.515	0.695	0.732
CORNIA	0.944	0.946	0.683	**0.742**	0.696	0.768	**0.621**	**0.658**	0.762	**0.800**
BLIINDS	0.927	0.930	0.662	0.697	**0.739**	**0.784**	0.503	0.538	0.738	0.764
NIQE	0.912	0.907	0.258	0.346	0.632	0.721	0.458	0.502	0.642	0.682
CNN-IQA	**0.946**	**0.948**	**0.722**	**0.750**	**0.854**	**0.878**	0.575	0.556	**0.820**	**0.832**
BRISQUE	0.940	0.943	0.600	0.654	0.738	0.758	0.602	0.636	0.741	0.768
DIIVINE	0.897	0.897	0.594	0.636	0.737	0.751	0.600	0.623	0.713	0.732
MVGCN	**0.946**	**0.947**	**0.688**	0.735	0.735	0.775	**0.622**	**0.646**	**0.771**	0.796

**Table 3.** SROCC for Database-independent experiments on MVGCN across multiple IQA databases. Rows: training dataset; Column: testing dataset. The overall performance is calculated for each training database.

Train/Test	LIVE	TID08	CSIQ	TID13	Overall
LIVE	-	0.927	0.905	0.922	0.919
TID08	0.841	-	0.678	0.876	0.813
CSIQ	0.787	0.801	-	0.765	0.785
TID13	0.839	0.955	0.662	-	0.862

**Table 4.** Aggregate results of Database-independent tests for various IQA models. The best two NR IQA models are boldfaced.

Model	Overall SROCC	Overall PLCC
SSEQ	0.810	0.833
CORNIA	**0.865**	**0.881**
BLIINDS	0.824	0.840
DIIVINE	0.840	0.844
BRISQUE	0.809	0.813
MVGCN	**0.854**	**0.863**

**Table 5.** Results of statistical significance test performed between SROCC values of different NR IQA algorithms across four databases. The elements in each cell correspond to the following databases (from left to right): LIVE, CSIQ, TID08 and LIVE Challenge. ‘1’ means that the row algorithm is statistically superior than the column algorithm with a confidence of 95%, ‘0’ signifies statistically worse and ‘-’ means statistical equivalence.

	SSEQ	BRISQUE	CORNIA	NIQE	DIIVINE	BLIINDS	MVGCN
SSEQ	----	0010	0–00	0111	−−10	0000	0000
BRISQUE	1101	----	0100	1111	1---	1–01	−−00
CORNIA	1–11	1011	----	1111	1–11	10–1	-0--
NIQE	1000	0000	0000	----	1000	0000	0000
DIIVINE	−−01	0---	0–00	0111	----	0–01	0–00
BLIINDS	1111	0–10	01–0	1111	1–10	----	0–00
MVGCN	1111	−−11	-1--	1111	1–11	1–11	----

**Table 6.** Comparison of median time taken per image to extract features by different NR IQA algorithms on a 4 GHz Quad-Core processor with 32 GBs of RAM. The median is computed over all distorted images from the LIVE database.

Algorithm	Time (in sec.)
SSEQ	0.77
CORNIA	2.10
BLIINDS	27.66
DIIVINE	9.28
BRISQUE	0.03
MVGCN	0.08

**Table 7.** The time complexity comparison between CORNIA and MVGCN-X-ray to extract features from an X-ray image of size 6329 × 5380 on a 4 GHz Quad-Core processor with 32 GBs of RAM.

Algorithm	Time (in sec.)
CORNIA	542.42
MVGCN-X-ray	9.61

**Table 8.** Feature summary for joint MSGCN(*m*), paired-products (*pp*) and joint paired-products (*j*) coefficients for the X-ray application. All features are extracted at two scales.

Feature ID	Feature Description	Computation Procedure
*m*_1_	shape	3-D MVGG fit to MSGCN coefficients
*m*_1_ - *m*_3_	eigenvalues of scale matrix	3-D MVGG fit to MSGCN coefficients
*pp*_1_ - *pp*_3_	shape, mean and right variance	AGGD fit to H pairwise coefficients
*pp*_4_ - *pp*_6_	shape, mean and right variance	AGGD fit to V pairwise coefficients
*j*_1_ - *j*_2_	eigenvalues of scale matrix	2-D MVGG fit to H and V pp coefficients

**Table 9.** Median log loss and AUC scores across 1000 train-test trials on different component-clutter combinations. The best two feature groups for each component-clutter category are boldfaced.

		IQIs		QUIX		MVGCN-X-ray		QUIX + IQIs		MVGCN + IQIs	
Component	Clutter type	log-loss	AUC	log-loss	AUC	log-loss	AUC	log-loss	AUC	log-loss	AUC
Power source	Clutter	**0.485**	**0.867**	0.489	0.831	0.500	0.820	**0.451**	**0.877**	0.503	0.863
Power source	Shield	0.528	0.863	**0.502**	**0.900**	**0.500**	**0.900**	0.526	0.880	0.506	0.888
Power source	Shield with Clutter	0.312	0.591	0.205	0.833	**0.178**	0.864	0.205	**0.900**	**0.176**	**0.864**
Power source	No Clutter	0.496	0.886	0.579	0.863	0.544	0.844	**0.480**	**0.895**	**0.470**	**0.889**
Detonator	Clutter	0.200	0.933	0.229	0.944	**0.191**	**0.962**	0.213	**0.938**	**0.199**	0.921
Detonator	Shield with Clutter	0.624	0.875	0.526	0.875	**0.337**	0.875	0.512	0.875	**0.340**	0.875
Detonator	No Clutter	0.455	0.944	0.395	0.944	0.407	0.944	**0.346**	0.944	**0.344**	0.944
Load	Clutter	0.163	0.944	0.137	0.944	**0.131**	0.944	0.134	0.944	**0.133**	0.944
Load	No Clutter	**0.505**	0.852	0.622	**0.883**	0.561	0.861	0.592	0.861	**0.559**	**0.889**
Switch	Clutter	0.318	0.932	0.275	**0.936**	**0.228**	**0.941**	0.249	0.927	**0.213**	0.932
Switch	Shield	0.369	0.932	**0.311**	**0.936**	0.350	**0.941**	**0.326**	0.927	0.351	0.932
Switch	No Clutter	0.414	**0.928**	0.506	0.872	0.476	0.872	**0.407**	0.907	**0.406**	**0.920**
Metal pipe	Clutter	**0.291**	**1.000**	0.397	0.125	0.346	0.500	0.304	**1.000**	**0.264**	0.875
Metal pipe	Shield	**0.394**	1.000	0.511	1.000	0.477	0.462	0.478	1.000	**0.453**	1.000
Metal pipe	Shield with Clutter	0.432	0.905	0.472	0.905	0.443	0.917	**0.363**	**0.952**	**0.375**	**0.952**
Weighted Average		0.402	0.881	0.414	0.874	0.382	0.870	**0.373**	**0.912**	**0.357**	**0.906**

## Footnotes

1 The maximum likelihood estimate of the mean of a generalized Gaussian density is given by: ${\text{argmin}}_{\mu }{\sum_{k=-K}^{K}\sum_{l=-L}^{L}\left|I_{k,l}(i,j)-\mu (i,j)\right|}^{\gamma }$. Optimizing over each block of size (2*K* + 1)×(2*L* + 1) of an image is computationally expensive, thus we instead use the sample mean of the coefficients given by (3), as used in [12].

3 Natural images constitute all photographic images taken of the world, but exclude computer-generated graphics.

4 Matlab’s imwrite function is used to control the degree of JPEG compression by specifying ‘Quality’ as argument in the imwrite function.

5 Computing the KL divergence between an empirical 5D joint distribution and its 5D Gaussian fit is computationally expensive for a large sample size, thus we resorted to only computing a bivariate joint distribution of immediate neighbors.

6 To be consistent with other algorithms, we utilized the single step framework of DIIVINE, directly mapping the features to MOS/DMOS while skipping the distortion identification stage.

7 Certain commercial equipment, instruments, or materials are identified inthis paper in order to specify the experimental procedure adequately. Such identification is not intended to imply recommendation or endorsement by the U.S. government, nor is it intended to imply that the materials or equipment identified are necessarily the best available for the purpose.

8 The size of the studied X-ray images is of the order of 6000 × 5000 pixels.

## Appendix

If **x** is distributed as a zero-mean MVGG (5), then the following properties follow [41]:

(8) $$\text{Var}(x)=\frac{2^{1/s}\Gamma \left(\frac{d+2}{2s}\right)}{d\Gamma \left(\frac{d}{2s}\right)}\Sigma$$

(9) $$\gamma_{1}(x)=0$$

(10) $$\gamma_{2}(x)=\frac{d^{2}\Gamma \left(\frac{d}{2s}\right)\Gamma \left(\frac{d+4}{2s}\right)}{\Gamma^{2}\left(\frac{d+2}{2s}\right)}-d(d+2)$$

where *γ*_1_ and *γ*_2_ denote multivariate skewness and kurtosis coefficients, respectively. A number of methods have been studied to estimate the shape (*s*) and scale (Σ) parameters of an MVGG distribution, including the recursive maximum likelihood estimation [61], the method of moments [41], and the Fisher Scoring algorithm [46]. We utilize the efficient and reliable moment-matching technique described in [41]. Specifically, given a set of *N* i.i.d. *d*-dimensional MVGG vectors, **x**_1_ … **x**_*N*_, compute the sample multivariate kurtosis coefficient as in [62]:

(11) $${\hat{\gamma }}_{2}\left(x_{1}\ldots x_{N}\right)=\frac{1}{N}\sum_{i=1}^{N}{\left(x_{i}^{T}S^{-1}x_{i}\right)}^{2}-d(d+2),$$

where **S** is the sample covariance. Equations (10) and (11) are used to estimate *s*, which is then substituted into Eq. (8) to compute Σ, where Var(**x**) is replaced by the sample covariance **S**.

## References

[1] Katsavounidis I Dynamic Optimizer - a perceptual video encoding optimization framework. https://medium.com/netflix-techblog/dynamic-optimizer-a-perceptual-video-encoding-optimization-framework-e19f1e3a277f.

[2] Wang Z; Bovik A; Evans B Blind measurement of blocking artifacts in images. Int. Conf. Image Proc, 2000, Vol. 3, pp. 981–984.

[3] Feng X; Allebach JP Measurement of ringing artifacts in JPEG images. Int’l Society for Opt. and Phot, 2006, Vol. 6076, p. 60760A.

[4] Sazzad ZP; Kawayoke Y; Horita Y No reference image quality assessment for JPEG2000 based on spatial features. Sig. Proc.: Image Comm 2008, 23, 257–268.

[5] Zhu X; Milanfar P A no-reference sharpness metric sensitive to blur and noise. Int’l Work. Qual. Multi. Exper, 2009.

[6] Charrier C; Lebrun G; Lezoray O A machine learning-based color image quality metric. Conf. Col. in Graph., Imag., Vision, 2006, Vol. 2006, pp. 251–256.

[7] Kim J; Zeng H; Ghadiyaram D; Lee S; Zhang L; Bovik AC Deep convolutional neural models for picture-quality prediction: Challenges and solutions to data-driven image quality assessment. IEEE Sig. Proc. Mag 2017, 34, 130–141.

[8] Kang L; Ye P; Li Y; Doermann D Convolutional neural networks for no-reference image quality assessment. Proceedings of the IEEE conference on computer vision and pattern recognition, 2014, pp. 1733–1740.

[9] Bosse S; Maniry D; Müller KR; Wiegand T; Samek W Deep neural networks for no-reference and full-reference image quality assessment. IEEE Tran. Image Process 2018, 27, 206–219. 10.1109/TIP.2017.2760518 29028191

[10] Mittal A; Soundararajan R; Bovik AC Making a “completely blind” image quality analyzer. IEEE Sig. Proc. Lett 2013, 20, 209–212.

[11] Zhang Y; Moorthy AK; Chandler DM; Bovik AC C-DIIVINE: No-reference image quality assessment based on local magnitude and phase statistics of natural scenes. Signal Proc.: Image Comm 2014, 29, 725–747.

[12] Mittal A; Moorthy AK; Bovik AC No-reference image quality assessment in the spatial domain. IEEE Trans. Image Process 2012, 21, 4695–4708. 22910118 10.1109/TIP.2012.2214050

[13] Moorthy AK; Bovik AC Blind image quality assessment: From natural scene statistics to perceptual quality. IEEE Trans. Image Process 2011, 20, 3350–3364. 21521667 10.1109/TIP.2011.2147325

[14] Gabarda S; Cristobal G Blind image quality assessment through anisotropy. J. Opt. Soc. Am. A 2007, 24, B42–B51. 10.1364/josaa.24.000b42 18059913

[15] Saad MA; Bovik AC; Charrier C Blind image quality assessment: A natural scene statistics approach in the DCT domain. IEEE Tran. Image Process 2012, 21, 3339–3352. 10.1109/TIP.2012.2191563 22453635

[16] Ballé J; Laparra V; Simoncelli EP Density modeling of images using a generalized normalization transformation. arXiv preprint arXiv:1511.06281 2015.

[17] Schwartz O; Simoncelli EP Natural signal statistics and sensory gain control. Nature neuroscience 2001, 4, 819. 11477428 10.1038/90526

[18] Wainwright M; Simoncelli E Scale mixtures of Gaussians and the statistics of natural images. Adv. Neur. Inf. Process. Sys 2000, 12, 855–861.

[19] Gupta P; Moorthy AK; Soundararajan R; Bovik AC Generalized Gaussian scale mixtures: A model for wavelet coefficients of natural images. Sign. Process.: Image Comm 2018, 66, 87–94.

[20] Deledalle CA; Parameswaran S; Nguyen TQ Image restoration with generalized Gaussian mixture model patch priors. arXiv preprint arXiv:1802.01458 2018.

[21] Goodall TR; Bovik AC; Paulter NG Tasking on natural statistics of infrared images. IEEE Trans. Image Proc 2016, 25, 65–79. 10.1109/TIP.2015.2496289 26540687

[22] Moreno-Villamarín DE; Benítez-Restrepo HD; Bovik AC Predicting the quality of fused long wave infrared and visible light images. IEEE Trans. Image Proc 2017, 26, 3479–3491. 10.1109/TIP.2017.2695898 28436873

[23] Gupta P; Glover J; Paulter NG Jr; Bovik AC Studying the Statistics of Natural X-ray Pictures. ASTM J. Test. Eval 2018.

[24] Ruderman DL; Bialek W Statistics of natural images: Scaling in the woods. Phys. Rev. Lett 1994, 73, 814. 10057546 10.1103/PhysRevLett.73.814

[25] Ruderman DL The statistics of natural images. Net. Comput. Neur. Sys 1994, 5, 517–548.

[26] Bovik AC Automatic prediction of perceptual image and video quality. Proc. IEEE 9 2013, 101, 2008–2024.

[27] Elad M; Aharon M Image denoising via sparse and redundant representations over learned dictionaries. IEEE Trans. Image Process 2006, 15, 3736–3745. 17153947 10.1109/tip.2006.881969

[28] Zhao YQ; Yang J Hyperspectral image denoising via sparse representation and low-rank constraint. IEEE Trans. Geosc. and Rem. Sen 2015, 53, 296–308.

[29] Gupta P; Bampis CG; Bovik AC Natural Scene Statistics for Noise Estimation IEEE Southwest Symp. on Image Anal. and Interp. (SSIAI), 2018, Las Vegas, NV, 4.

[30] Mairal J; Elad M; Sapiro G Sparse representation for color image restoration. IEEE Trans. Image Process, 2008, 17, 53–69. 18229804 10.1109/tip.2007.911828

[31] Liu J; Huang TZ; Selesnick IW; Lv XG; Chen PY Image restoration using total variation with overlapping group sparsity. Information Sciences 2015, 295, 232–246.

[32] Wright J; Yang AY; Ganesh A; Sastry SS; Ma Y Robust face recognition via sparse representation. IEEE Trans. on Pattern Anal. Machine Intell, 2009, 31, 210–227. 10.1109/TPAMI.2008.79 19110489

[33] Jiang X; Lai J Sparse and dense hybrid representation via dictionary decomposition for face recognition. IEEE Trans. on Pattern Anal. Machine Intell, 2015, 37, 1067–1079. 10.1109/TPAMI.2014.2359453 26353329

[34] Agarwal S; Roth D Learning a sparse representation for object detection. Eur. Conf. on Comp. Vision 2006, pp. 97–101.

[35] Yokoya N; Iwasaki A Object detection based on sparse representation and Hough voting for optical remote sensing imagery. IEEE Jnl Sel. Top. App. Earth Obs. Remote Sensing 2015, 8, 2053–2062.

[36] Mairal J; Bach F; Ponce J; Sapiro G; Zisserman A Discriminative learned dictionaries for local image analysis. Comp. Vision Pattern Recogn, 2008.

[37] Minaee S; Abdolrashidi A; Wang Y Screen content image segmentation using sparse-smooth decomposition. Asilomar Conf. Sig., Sys. and Comp„ 2015, pp. 1202–1206.

[38] Sharifi K; Leon-Garcia A Estimation of shape parameter for generalized Gaussian distributions in subband decompositions of video. IEEE Trans. Cir. Sys. Vid. Technol 1995, 5, 52–56.

[39] Wainwright MJ; Schwartz O; Simoncelli EP Natural image statistics and divisive normalization: modeling nonlinearities and adaptation in cortical neurons. Statistical Theories of the Brain 2002, pp. 203–222.

[40] Sheikh HR; Sabir MF; Bovik AC A Statistical Evaluation of Recent Full Reference Image Quality Assessment Algorithms. IEEE Trans. Image Process 2006, 15, 3440–3451. 17076403 10.1109/tip.2006.881959

[41] Gómez E; Gomez-Viilegas M; Marin J A multivariate generalization of the power exponential family of distributions. Comm. Stat.-Theory Meth 1998, 27, 589–600.

[42] Kwitt R; Meerwald P; Uhl A Color-image watermarking using multivariate power-exponential distribution. IEEE Int’l Conf. Image Process., 2009 2010, pp. 4245–4248.

[43] Coban M; Mersereau R Adaptive subband video coding using bivariate generalized Gaussian distribution model IEEE Intl. Conf. Img. Proc, 2002 IEEE, 2002, Vol. 4, pp. 1990–1993.

[44] Verdoolaege G; De Backer S; Scheunders P Multiscale colour texture retrieval using the geodesic distance between multivariate generalized Gaussian models. IEEE Int’l Conf. Image Process. IEEE, 2008, pp. 169–172.

[45] Omari M; Abdelouahad AA; El Hassouni M; Cherifi H Color image quality assessment measure using multivariate generalized Gaussian distribution. Int’l Conf. Sig. Image Tech. Int. Based Sys, pp. 195–200.

[46] Verdoolaege G; Scheunders P Geodesics on the manifold of multivariate generalized Gaussian distributions with an application to multicomponent texture discrimination. Int’l. J. Comp. Vis 2011, 95, 265.

[47] Ma K; Duanmu Z; Wu Q; Wang Z; Yong H; Li H; Zhang L Waterloo exploration database: New challenges for image quality assessment models. IEEE Tran. Image Process 2017, 26, 1004–1016. 10.1109/TIP.2016.2631888 27893392

[48] Martin D; Fowlkes C; Tal D; Malik J A Database of Human Segmented Natural Images and its Application to Evaluating Segmentation Algorithms and Measuring Ecological Statistics. Int’l Conf. Comp. Vis, 2001, Vol. 2, pp. 416–423.

[49] Lasmar NE; Stitou Y; Berthoumieu Y Multiscale skewed heavy tailed model for texture analysis. IEEE Int’l Conf. Image Proc. (ICIP), 2009.

[50] Liu L; Liu B; Huang H; Bovik AC No-reference image quality assessment based on spatial and spectral entropies. Signal Proc.: Image Comm 2014, 29, 856–863.

[51] Ye P; Kumar J; Kang L; Doermann D Unsupervised feature learning framework for no-reference image quality assessment. IEEE Conf. on Comp. Vision Pattern Recogn, 2012, pp. 1098–1105.

[52] Wang Z; Simoncelli EP; Bovik AC Multi-scale structural similarity for image quality assessment. Proc. IEEE Asilomar Conf. on Signals, Systems, and Computers, (Asilomar) 2003.

[53] Ponomarenko N; Lukin V; Zelensky A; Egiazarian K; Carli M; Battisti F TID2008-a database for evaluation of full-reference visual quality assessment metrics. Adv. Mod. Radioelectronics 2009, 10, 30–45.

[54] Larson EC; Chandler DM Most apparent distortion: full-reference image quality assessment and the role of strategy. J. Elect. Imag 2010, 19, 011006–011006.

[55] Ghadiyaram D; Bovik AC Massive online crowdsourced study of subjective and objective picture quality. IEEE Trans. Image Process 2016, 25, 372–387. 26571530 10.1109/TIP.2015.2500021

[56] Corey DM; Dunlap WP; Burke MJ Averaging correlations: Expected values and bias in combined Pearson rs and Fisher’s z transformations. J. Gen. Psych 1998, 125, 245–261.

[57] Gupta P; Sinno Z; Glover J; Paulter NG Jr; Bovik AC Predicting detection performance on security X-ray images as a function of image quality. IEEE Trans. Image Process submitted. 10.1109/TIP.2019.2896488 PMC7433314 30714919

[58] Toolkit X-Ray. http://www.xraytoolkit.com/. Accessed: 2018-06-01.

[59] Glover JL; Gupta P; Bovik AC; Paulter NG Measuring and modeling the detectability of IED components in X-ray images as a function of image quality **in preparation**.

[60] American national standard for the performance of portable transmission x-ray systems for use in improvised explosive device and hazardous device detection. IEEE/ANSI-N42.55–2013 Approved 12 2013.

[61] Pascal F; Bombrun L; Tourneret JY; Berthoumieu Y Parameter Estimation For Multivariate Generalized Gaussian Distributions. IEEE Trans. Sig. Proc 2013, 61, 5960–5971.

[62] Mardia KV Applications of some measures of multivariate skewness and kurtosis in testing normality and robustness studies. The Indian J. Stat 1974, 36, 115–128.

